# Exploiting serological data to understand the epidemiology of bluetongue virus serotypes circulating in Libya

**DOI:** 10.1002/vms3.136

**Published:** 2018-11-23

**Authors:** Abduslam S. Mahmoud, Giovanni Savini, Massimo Spedicato, Federica Monaco, Irene Carmine, Alessio Lorusso, Tolari Francesco, Maurizio Mazzei, Mario Forzan, Ibrahim Eldaghayes, Abdunaser Dayhum

**Affiliations:** ^1^ L'Istituto Zooprofilattico Sperimentale dell'Abruzzo e del Molise “Giuseppe Caporale” (IZSAM) Teramo Italy; ^2^ Department of Veterinary Science University of Pisa Pisa Italy; ^3^ Faculty of Veterinary Medicine University of Tripoli Tripoli Libya; ^4^ National Center of Animal Health Tripoli Libya

**Keywords:** BT, Novel BTV serotypes, BTV‐3, BTV‐26, Sero‐prevalence, Libya

## Abstract

The epidemiological patterns of Bluetongue (BT) in North Africa and Mediterranean Basin (MB) dramatically changed by emergence of subsequent episodes of novel bluetongue virus (BTV) serotypes with highly pathogenic indexes and socio‐economic impacts. The objective of the study was to investigate the sero‐prevalence and serotype distribution of BTV in Libya. During 2015‐2016, a total of 826 serum samples were collected from domestic ruminants in Libya. All sera were assayed by competitive enzyme‐linked immunosorbent assays (c‐ELISA). C‐Elisa‐positive samples (43.3%; 173/400) were further analyzed by virus neutralization assay to identify BTV serotypes and determine the antibody titre of positive samples. An overall BTV sero‐prevalence was 48.4% (95% CI: 45.0%‐51.8%). Neutralizing antibodies were detected against the following BTV serotypes namely: BTV‐1, BTV‐2, BTV‐3, BTV‐4, BTV‐9 and BTV‐26. While BTV‐1, BTV‐2, BTV‐4 and BTV‐9 circulation was unsurprising as they have been responsible of the last year outbreaks in Northern African Countries, the detection of BTV‐3 and BTV‐26 was definitely new and concerning for the animal health of the countries facing the Mediterranean Basin. It is crucial that European and Northern African authorities collaborate in organizing common surveillance programmes to early detect novel strains or emerging serotypes in order to set up proper preventive measures, and, in case, develop specific vaccines and plan coordinated vaccination campaigns.

## Introduction

Bluetongue (BT) is an infectious, vector‐borne viral disease that affects wild and domestic ruminants. The disease is caused by bluetongue virus (BTV), a member of the genus *Orbivirus* within the family *Reoviridae*. Up to now, 27 serotypes of BTV are officially recognized (Maan *et al*. 2011a,b; Zientara *et al*. 2014), and many others have been recently described (Savini *et al*. 2017). While the epidemiologic features of BTV 1‐24 infections are similar in that they are all spread predominantly by *Culicoides*, there is uncertainty regarding the exclusive role of midges in the transmission of BTV‐25, BTV‐26 and BTV‐27 (Vogtlin *et al*. 2013; Batten *et al*. 2014; Maclachlan *et al*. 2015). Horizontal transmission has been demonstrated for BTV‐26 and 27 and hypothesized for the other new serotypes (Batten *et al*. 2014; Bréard *et al*. 2017).

In the last couple of decades, countries facing the Mediterranean Basin and particularly those belonging to the Maghreb region have been the target of several BTV incursions involving different strains of BTV‐1, BTV‐2, BTV‐3 and BTV‐4 (Hammami 2004; Nomikou *et al*. 2009; Lorusso *et al*. 2014; Sghaier *et al*. 2017). Libya is a Northern African country located in the Mediterranean Region. Due to the socio‐political instability of the country and vulnerability of the quarantine measures, many transboundary animal diseases such as Foot‐Mouth‐Disease (Eldaghayes *et al*. 2017), peste des petit ruminants (Dayhum *et al*. 2018), and Brucellosis (FAO, 2013), emerged and/or re‐emerged. BT is known to be present in Libya but apart from a report on a BTV‐1 strain isolated and identified in 2007 in the Green Mountain branch (Eastern Libya) (LIB2007/06) and a BTV‐9 Libyan strain (LIB2008/08) isolated in 2008 from sheep showing BT clinical signs (Nomikou *et al*. 2009), no information is available on the sero‐prevalence and distribution of these serotypes in the country. This study is the first investigation on the sero‐prevalence and serotype distribution of BTV in Libya.

## Materials and methods

**Figure 1:**
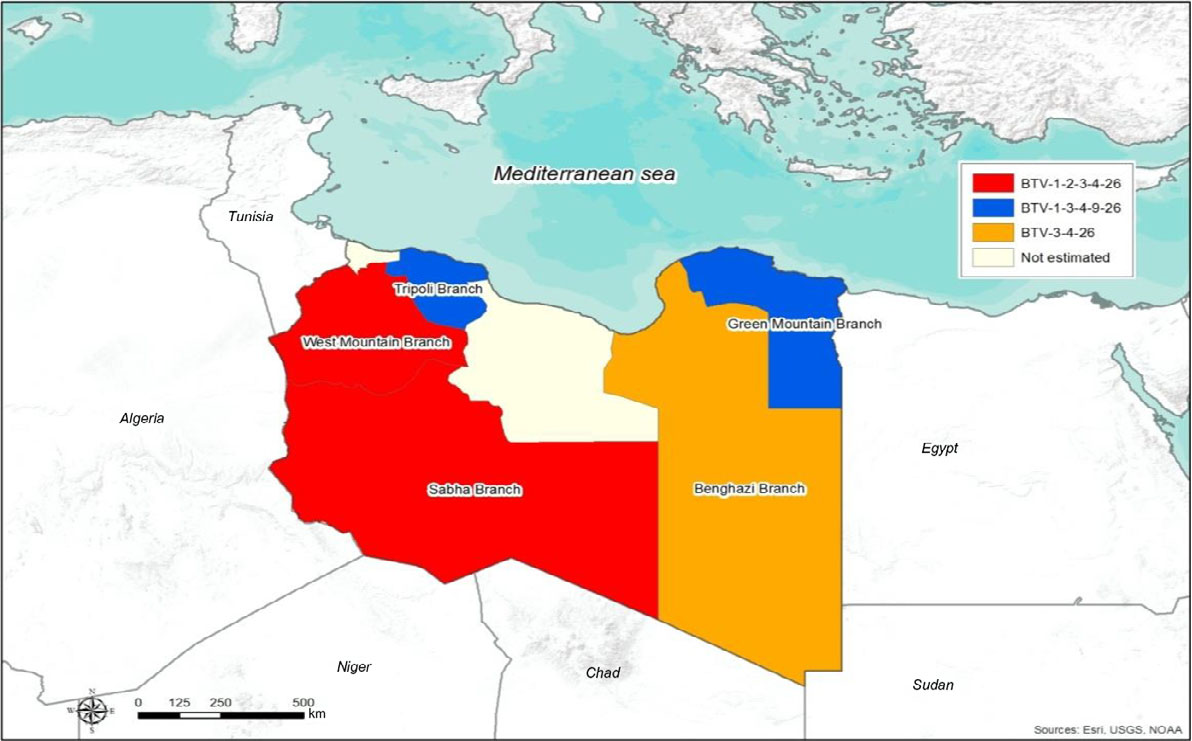
The spatial distribution of Bluetongue virus serotypes according to Libyan branches.

During 2015–2016, a total of 826 serum samples were collected from 152 cattle, 542 sheep and 132 goats of 96 farms representative of the eleven provinces distributed in five NCAH branches of Green mountain, Benghazi, Tripoli, West mountain and Sabha (Fig. 1 and Table 1). In each farm, a maximum of 10 serum samples were gathered regardless the herd size. The sampling was designed to detect the minimum prevalence of 3% between herds with 95% confidence and to detect the minimum prevalence of 25% within the herd with 95% confidence. Due to the national socio‐political situation, the samples were collected only where the sampling activity was feasible. No vaccination programme against BT is practised in Libya and all samples were collected from animals which had never experienced vaccination against BTV.

**Table 1:** Number of samples and Bluetongue virus sero‐prevalence in Libyan branches

Branches	Tested Total	Negative	Positive	Seroprev	95% LCL	95% UCL
West Mountain Branch	305	143	162	53.11%	47.510%	58.720%
Tripoli Branch	60	38	22	36.67%	24.470%	48.860%
Benghazi Branch	249	134	115	46.2%	43.830%	55.170%
Green Mountain Branch	171	101	70	40.9%	33.570%	48.310%
Sabha Branch	41	10	31	75.61%	62.460%	88.750%
Total	826	426	400	48.43%	45.02%	51.83%

LCL, lower confidence limit; UCL, upper confidence limit.

Serum samples were stored at −20°C and then shipped to the OIE and National Reference Laboratory for BT, Teramo, Italy (IZS TE) where the serological tests were performed. Serum samples were tested by competitive enzyme‐linked immunosorbent assay (c‐ELISA) for the detection of antibodies against BTV, by using the BT antibody test kit c‐ELISA rec VP7 (IZSAM, Teramo, Italy). (Tittarelli 2014).

C‐ELISA‐positive sera were further analyzed by virus neutralization assay (VNT) to identify BTV serotypes and determine the antibody titre according to OIE Manual (OIE, 2014).

The influence of variables like sex, species, age and geographic origin of the animals on the prevalence was also assessed. Animals were divided into two groups according to age: Group I (7–24 months) and Group II (more than 24 months). Statistical analysis was performed using XLSTAT. For each proportion, the prevalence and 95% confidence intervals (CI) were calculated using the Bayesian approach of Beta distribution. A chi‐square test was used to evaluate the probability that differences observed among sero‐prevalences of infection. Association between the outcome variables and its potential risk factors were screened in a univariable analysis using chi‐square test. A *P* < 0.05 was considered to be significant.

## Results

In the present study, the overall sero‐prevalence of BTV in cattle and small ruminants was 48.4% (400/826; 95% CI: 45.0–51.8%). The sero‐prevalence was higher (*P* = 0.0235) in sheep and goats (339/674; 50.3%; 95% CI: 46.5–54.1%;) compared with cattle (61/152; 40.1%; 95% CI: 32.7–48.1%) (Table 2).

**Table 2:** Bluetongue virus sero‐prevalence in Libya: results of univariate analysis of the independent variables which include, animal species, sex, age group and Libyan branches

Risk factors	Animal tested	Animal affected (%)	df	*X* ^2^	*P*‐value
Species	826		1	5.1	0.0235
Small ruminants	674	50.3%			
2Cattle	152	40.1%			
Sex			1	17.2	0.00004
Male	69	23.2%			
Female	716	49.3%			
Age group			1	24.2	0.00001
Young (7 ‐23 months)	321	37.7%			
Adult (equal or older than 24 months)	505	55.2%			
Libyan Branches			1	Fisher's exact test	0.025
Sabha branch (Southern region)	41	75.61%			0.0003
All other branches	785	47.01%			

Df, degree freedom.

In the five NCAH branches, the overall sero‐prevalence was not uniformly (*P* = 0.025) distributed and a significantly (*P* = 0.0003). Higher sero‐prevalence was recorded in Sabha branch (Southern region) (31/41; 75.6%; 95% CI: 60.5–86.1%;); conversely, the other branches showed similar overall and species sero‐prevalences (Tables 1, 2 and Fig. 1).

**Figure 2:**
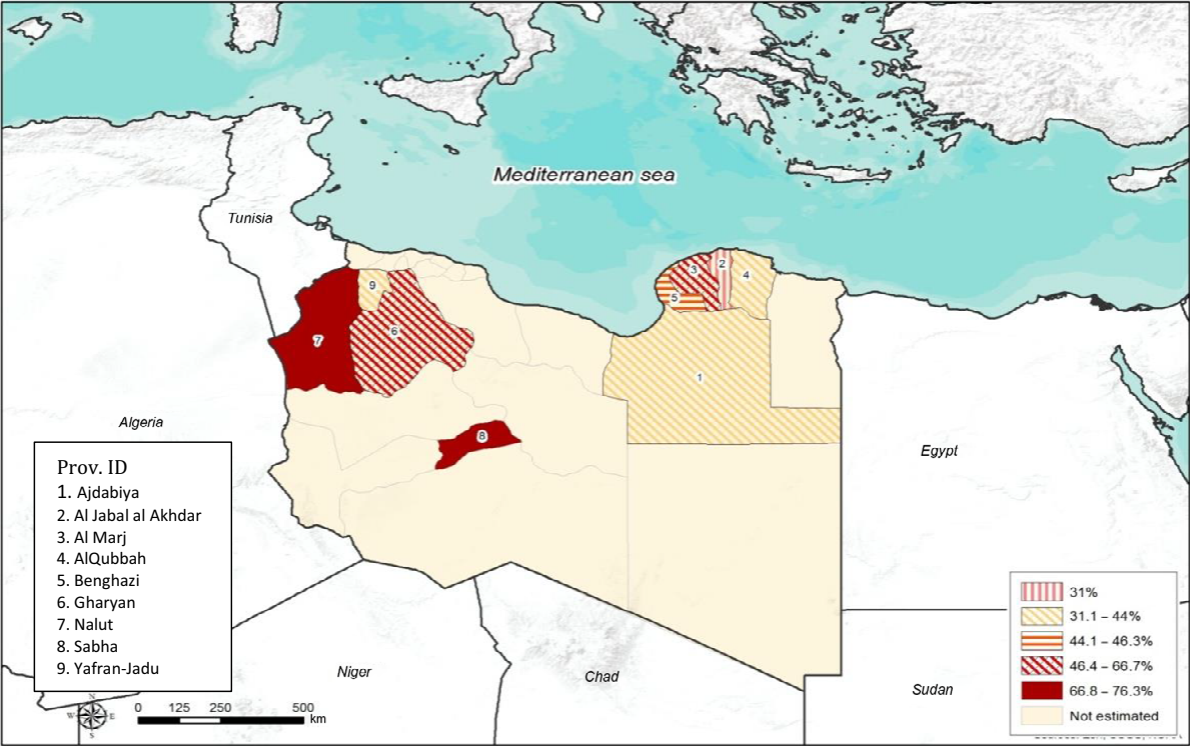
Sero‐prevalence based on c‐ELISA and spatial distribution of Bluetongue virus in small ruminants according to Libyan provinces.

**Figure 3:**
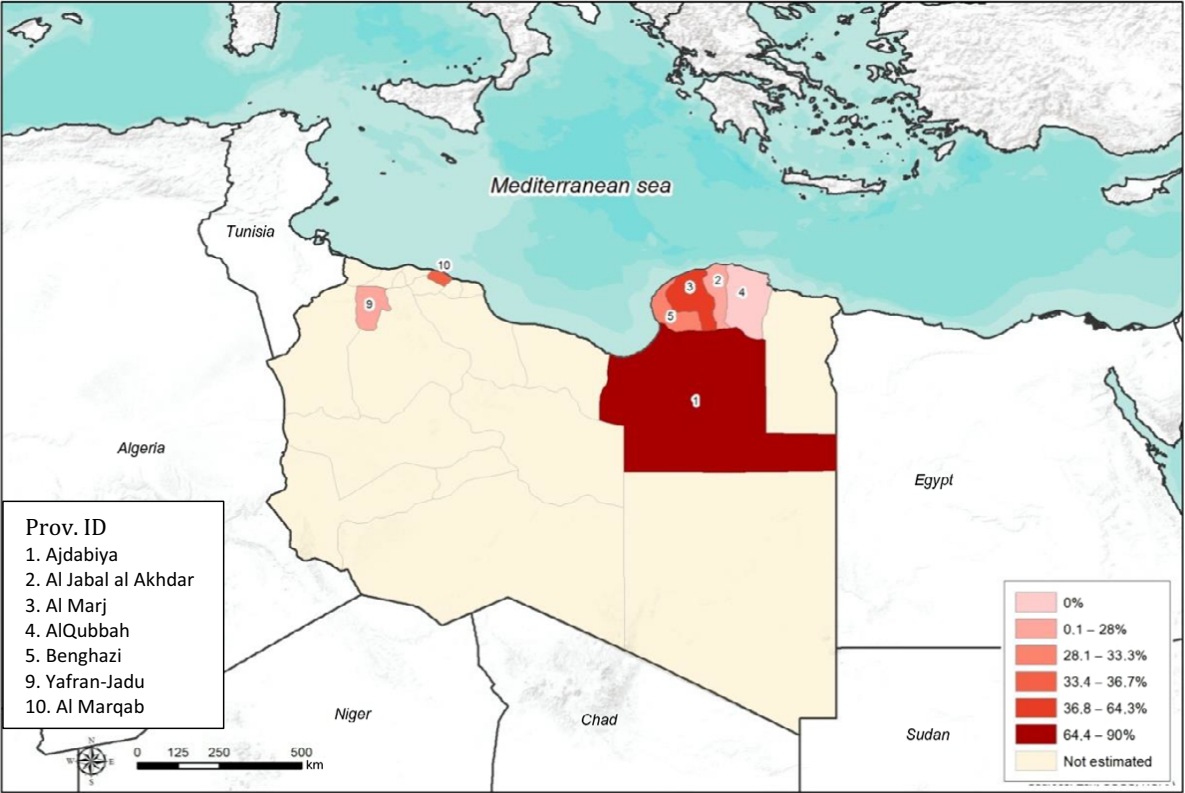
Sero‐prevalence based on c‐ELISA and spatial distribution of Bluetongue virus in cattle according to Libyan provinces.

Similarly, significant differences were found between small (*P* = 0.00001) and large (*P* = 0.0011) ruminant prevalence values in Libyan provinces. In the small ruminants, the highest prevalence values were found in Nalut (Western region) and Sabha (Southern region) provinces with 76.3% (45/59; 95% CI: 64.0–85.3%) and 75.6% (31/41; 95% CI: 60.5–86.1%), respectively, while the lowest values were recorded in Al Jabal al Akhdar (Eastern region) and Yafran‐Jadu (Western region) provinces with 31.0% (26/84; 95% CI: 22.8–41.5%) and 34.4% (44/128; 95% CI: 26.7–43.0%), respectively (Fig. 2). In cattle, the highest sero‐prevalence was reported in Ajdabiya province (Eastern region) (9/10; 90%; 95% CI: 58.7–97.7%), while the lowest sero‐prevalence was found in Yafran‐Jadu province (Western region) (1/10; 10%; 95% CI: 2.3–41.3%) (Fig. 3).

Sex (*P* = 0.00001) and age (*P* = 0.00004) significantly influenced the BTV sero‐prevalence in Libya. BTV infection rate was significantly higher in females (353/716; 49.3%; 95% CI: 40.9–59.1%) and in group II (animals more than 24 months) (279/505; 55.2%; 95% CI:50.9–59.5%) (Table 2).

Forty‐three per cent (173/400) of the positive samples were further analyzed by virus neutralization assay to identify BTV serotypes. Of the 173 samples tested by VNT for all serotypes except BTV‐25, 44 had neutralizing antibodies with titres ranging from 1:10 to more than 1:80 against one or more of the following serotypes: BTV‐1, BTV‐2, BTV‐3, BTV‐4, BTV‐9 and BTV‐26 (Fig. 1).

Twenty‐nine sera had titres against one serotype only, (three for BTV‐1, five for BTV‐2, four for BTV‐3, thirteen for BTV‐4 and four for BTV‐26) (Table 3). Mixed infections (presence of different serotype‐specific neutralizing antibodies) were detected in the other fifteen sera as follows: Nine with double infection; four with triple infection; one with quadruple infection; and one with quintuple infection, as seen in Table 3.

**Table 3:** Bluetongue virus serotypes circulating in Libya

Serotype	Single inf.	Double inf.	Triple inf.	Quadruple inf.	Quintuple inf.
BTV‐1	3	2 (BTV‐26)	2 (BTV‐3 + BTV‐26)	1 (BTV‐2 + BTV 3 + BTV‐4)	1 (BTV‐3 + BTV‐4 + BTV‐9 + BTV26)
BTV‐2	5	–	–	–	–
BTV‐3	4	1 (BTV‐26), 2 (BTV‐4), 1 (BTV‐9)	2 (BTV‐4 + BTV‐26)	–	–
BTV‐4	13	2 (BTV‐26)	–	–	–
BTV‐9	–	1 (BTV‐26)	–	–	–
BTV‐26	4	–	–	–	–
Total	29	9	4	1	1

The presence of serotype‐specific neutralizing antibodies implies that all detected serotypes have circulated or are still circulating in the Northwest region of Libya. Conversely, BTV‐2 was not found in the Eastern region, while BTV‐1 and BTV‐9 were not detected in the Southern region, Sabha province.

## Discussion

A multivariate logistic regression analysis would have probably given a more appropriate answer on the influence on the BTV prevalence of each of the variables considered in this study, but the high number factors analyzed, and the relative exiguous number of samples did not allow us to use this more suitable analytical approach. The results of this study however are still original and interesting offering precious and valuable information for the European and northern African scientific communities.

This study is the first investigation on the sero‐prevalence and serotype distribution of BTV in Libya. In line with other Northern African countries (Mahmoud & Khafagi 2014), the obtained results (48.4%; 95% CI: 45.0–51.8%) confirmed that, at least in the surveyed regions and provinces, BT is endemic in Libya. Moreover, the sero‐prevalence values observed in the two groups of age included in this study clearly indicate that this endemic condition in Libya is stable. In fact, for both small and large ruminants, the sero‐prevalence values in the Group II were significantly different from young group. The differences between Group I and Group II were probably due to the fact that as animals mature, the chance of becoming infected with BTV increases because they are exposed to more BTV‐infected vector periods. Endemic stability is a situation in which all factors influencing disease occurrence are relatively stable, resulting in little fluctuation in disease incidence over time; clinical disease is rare in spite of a high incidence of infection within a population. Interestingly, although the sampling included the Sabha province only, and therefore could not be representative of the entire south region, the highest (*P* = 0.0003) prevalence values (75.6%; 95% CI: 51.1–87.1%) were observed in the Southern part of Libya where animal movements across the borders either transitional or illegal are recurrent and, thus, the risk of importing animals infected with different pathogens from the Southern borders is high (Dayhum *et al*. 2018).

In line with other similar studies indicating a higher sero‐prevalence in females than males (Elhassan *et al*. 2014), the significant difference between sex (*P* = 0.011) found in this survey could be explained by the fact that farmers sell the bulls after weaning resulting in higher numbers of older females than older males.

Concerning VNT, only 173 samples were selected out of 400 positive samples mainly for two specific reasons: the first was that when there are many positive samples in the same farm or at the same area, we did not send all positive samples for further investigation as we would not expect to find different serotypes circulating in the same farm and/or area, and the second reason was to reduce the cost and to save time as VNT is more expensive and time consuming, so hence only 43% of positive samples were sent for further testing by VNT. We would not expect to find different results for the remaining positive samples.

As expected, neutralizing antibodies were detected in only 25% (44/173) of the c‐ELISA positive samples. It is known that c‐ELISA is more sensitive and could detect antibodies for a longer period than VNT (Afshar 1994). Neutralizing antibodies against six different serotypes namely BTV‐1, BTV‐2, BTV‐3, BTV‐4, BTV‐9 and BTV‐26 have been detected. While BTV‐1, BTV‐2, BTV‐4 and BTV‐9 circulation was unsurprising as they have been responsible of the last year outbreaks in Northern African Countries (Nomikou *et al*. 2009; Lorusso *et al*. 2014), the detection of BTV‐3 and BTV‐26, if not totally unexpected, was definitely concerning. Unfortunately, as a serological survey, this study is unable to add more information on the current circulation of these strains in Libya and/or their genome characteristic. BTV‐9 antibodies were also detected in animals from two Libyan branches only, Benghazi and Tripoli (Northern Libya). The evolution of this serotype in Northern Africa seems to differ from the other serotypes which have circulated or are circulating in Northern Africa as the BTV‐9 Libyan strain did not expand neither to the neighbouring nor to the South European countries. The same cannot be said for BTV‐3 as it can be considered an emerging strain/serotype in Northern Africa. Although recorded in Sudan (Elfatih *et al*. 1987), BTV‐3 has never been reported in Northern Africa before 2016 when high case fatality rates caused by BTV‐3 were observed in sheep in Tunisia (Sghaier *et al*. 2017). As previously said, illegal trade and free livestock movements or nomadic and transhumant pastoralist activities present and common in the Southern regions might have facilitated the introduction of this emerging serotype already reported in Sudan (Elfatih *et al*. 1987). High transitional animal movement where huge number of imported animals crossing the borders and nomadism practices might have also enabled the incursion of BTV‐26 in Libya from the Southern bordering countries. This is the first time that BTV‐26 circulation was demonstrated in Libya where it has been detected in animals from all branches included in this survey.

BTV‐26 is a recently described serotype. It was isolated in Kuwait from sheep (virus isolate KUW2010/02) (Maan *et al*. 2011a) and in Mauritania from cattle (Lorusso *et al*. 2016). The particularity of this serotype is that in the absence of the vector insects it can be horizontally transmitted to uninfected goats, which subsequently seroconvert (Batten *et al*. 2013, 2014). The presence of BTV‐26 in a country is not of great concern today as this serotype generally does not cause neither clinical signs in infected animals nor restriction measures in infected countries. However, more than one‐third (34%) of the animals with BTV neutralizing titres had antibodies against two or more serotypes. Although based on serology only it is not possible to say whether these animals were contemporaneously infected with two or more BTV serotypes, the chance that co‐infection might have occurred and/or occur in Libya is not negligible. Interestingly, most (78.5%) of the possible co‐infections found in this study included BTV‐26 and, thus, the formation of a reassortment strain with the pathogenic characteristics of the BTV‐1, BTV‐2, BTV‐3, BTV‐4 and BTV‐9 strains and capable of transmitting horizontally, which is a peculiarity of the BTV‐26, is far from being a remote event.

Considering the origin of the previous BTV European incursions and that at the moment inactivated vaccines against these serotypes are not available, BTV‐3 and BTV‐26 in Northern Africa in general, and in Libya, in particular, represent a great concern for the animal health of the countries facing the Mediterranean basin. It is crucial that European and Northern African authorities collaborate in organizing common surveillance programmes to early detect novel strains or emerging serotypes in order to set up proper preventive measures, and, in case, develop specific vaccines and plan coordinated vaccination campaigns.

## Source of funding

The authors would like to express their gratitude to Libyan Ministry of High Education for the scholarship of Abdusalam Mahmoud for Ph. D degree and to the staff of the National Reference Centre of Istituto Zooprofilattico Sperimentale dell’ Abruzzo e del Molise G Caporale (IZSAM), Teramo, Italy, for providing fund and samples testing.

## Conflict of interest

The Authors declare that there is no conflict of interest.

## Ethical statement

The authors confirm that the ethical policies of the journal, as noted on the journal's author guidelines page, have been adhered to. In this study, verbal consent of farm owners was obtained prior to the collection of blood samples from their animals. Animals were used just once for jugular venipuncture by professional veterinary technologists. So, no ethical approval was required as non‐invasive procedure was carried out in this study.

## Contributions

Study Design: Abdusalam Mahmoud, Giovanni Savini, Federica Monaco, Francesco Tolar, Abdunaser Dayhum. Laboratory Work: Abdusalam Mahmoud, Giovanni Savini, Federica Monaco. Statistical Analysis: Abdusalam Mahmoud, Abdunaser Dayhum. Draft Manuscript Preparation: All authors. Manuscript Revision and Approval: All authors.

## References

[vms3136-bib-0001] Afshar A. (1994) Bluetongue: laboratory diagnosis. Comparative Immunology, Microbiology and Infectious Diseases 17, 221–242. 10.1016/0147-9571(94)90045-0 8001347

[vms3136-bib-0002] Batten C.A. , Henstock M.R. , Steedman H.M. , Waddington S. , Edwards L. & Oura C.A. (2013) Bluetongue virus serotype 26: infection kinetics, pathogenesis and possible contact transmission in goats. Veterinary Microbiology 162, 62–67. 22986055 10.1016/j.vetmic.2012.08.014

[vms3136-bib-0003] Batten C. , Darpel K. , Henstock M. , Fay P. , Veronesi E. , Gubbins S. *et al* (2014) Evidence for transmission of Bluetongue virus serotype 26 through direct contact. PLoS ONE 9, e96049. 24797910 10.1371/journal.pone.0096049 PMC4010411

[vms3136-bib-0004] Bréard E. , Schulz C. , Sailleau C. , Bernelin‐Cottet C. , Viarouge C. , Vitour D. *et al* (2017) Bluetongue virus serotype 27: experimental infection of goats, sheep and cattle with three BTV‐27 variants reveal atypical characteristics and likely direct contact transmission BTV‐27 between goats. Transboundary and Emerging Diseases 65, e251–e263. [Epub ahead of print] 29243405 10.1111/tbed.12780

[vms3136-bib-0005] Dayhum A. , Sharif M. , Eldaghayes I. , Kammon A. , Calistri P. , Danzetta M.L. *et al* (2018). Sero‐prevalence and epidemiology of peste des petits ruminants in Libya. Transboundary and Emerging Diseases Journal 65, e48–e54. 10.1111/tbed.12670 28703449

[vms3136-bib-0006] Eldaghayes I. , Dayhum A. , Kammon A. , Sharif M. , Ferrari G. , Bartels C. *et al* (2017) Exploiting serological data to understand the epidemiology of foot‐and‐mouth disease virus serotypes circulating in Libya. Open Veterinary Journal 7, 1–11. 28180094 10.4314/ovj.v7i1.1 PMC5283054

[vms3136-bib-0007] Elfatih M. , Mohammed H. & Taylor W.P. (1987) Infection with bluetongue and related orbiviruses in the Sudan detected by the study of sentinel calf herds. Epidemiology and Infection 99, 533–545. 2824226 10.1017/s0950268800068035 PMC2249265

[vms3136-bib-0008] Elhassan A.M. , Fadol M.A. , Hussein A.R . (2014) Seroprevalence of bluetongue virus in dairy herds with reproductive problems in Sudan. ISRN Veterinary Science 2014, 595724, 4 pages. 25002977 10.1155/2014/595724 PMC4060552

[vms3136-bib-0009] FAO , Sub‐Regional Office, North Africa. (2013). Transboundary animal diseases: diseases with strong social and economic impact. 3rd Quarter/2013. www.fao.org

[vms3136-bib-0010] Hammami S. (2004) North Africa: a regional overview of bluetongue virus, vectors, surveillance and unique features. Veterinaria Italiana 40, 43–46. 20419633

[vms3136-bib-0011] Lorusso A. , Sghaier S. , Ancora M. , Marcacci M. , Di Gennaro A. , Portanti O. *et al* (2014) Molecular epidemiology of bluetongue virus serotype 1 circulating in Italy and its connection with northern Africa. Infection, Genetics and Evolution 28, 144–149. 10.1016/j.meegid.2014.09.014 25239524

[vms3136-bib-0012] Lorusso A. , Baba D. , Spedicato M. , Teodori L. , Bonfini B. , Marcacci M. *et al* (2016) Bluetongue virus surveillance in the islamic republic of mauritania: is serotype 26 circulating among cattle and dromedaries? Infection, Genetics and Evolution 40, 109–112. 10.1016/j.meegid.2016.02.036 26932578

[vms3136-bib-0013] Maan S. , Maan N.S. , Nomikou K. , Batten C. , Antony F. , Belaganahalli M.N. *et al* (2011a) Novel bluetongue virus serotype from Kuwait. Emerging Infectious Diseases 17, 886–889. 21529403 10.3201/eid1705.101742 PMC3321788

[vms3136-bib-0014] Maan S. , Maan N.S. , Nomikou K. , Veronesi E. , Bachanek‐Bankowska K. , Belaganahalli M.N. *et al* (2011b) Complete genome characterization of a novel 26th bluetongue virus serotype from Kuwait. PLoS ONE 6, e26147. 22031822 10.1371/journal.pone.0026147 PMC3198726

[vms3136-bib-0015] Maclachlan N.J. , Mayo C.E. , Daniels P.W. , Savini G. , Zientara S. & Gibbs E.P.J. (2015) Bluetongue. Revue Scientifique et Technique (Office International des Épizooties) 34, 329–340. 10.20506/rst.34.2.2360 26601438

[vms3136-bib-0016] Mahmoud M.A. & Khafagi H.M. (2014) Seroprevalence of bluetongue in sheep and goats in Egypt. Veterinary World 10, 1161–1166. EISSN: 2231‐0916.

[vms3136-bib-0017] Nomikou K. , Maan S. , Maan N.S. & Mertens P.P.C. (2009) Molecular epidemiology of Bluetongue virus serotype 9 in the mediterranean region. Revue D’élevage et de Médecine Vétérinaire des Pays Tropicaux 62, 81–180.

[vms3136-bib-0018] OIE (Office International des Epizooties). (2014) Terrestrial Manual. Chapter 2.1.3. Bluetongue (Infection with Bluetongue virus). Available at: http://www.oie.int/fileadmin/Home/eng/Health_standards/tahm/2.01.03_BLUETONGUE.pdf

[vms3136-bib-0020] Savini G. , Poggioni G. , Meloni G. , Marcacci M. , Di Domenico M. , Rocchigiani A.M. *et al* (2017) Novel putative bluetongue virus in healthy goats from Sardinia, Italy. Infection, Genetics and Evolution 51, 108–117. 10.1016/j.meegid.2017.03.021 28341545

[vms3136-bib-0021] Sghaier S. , Lorusso A. , Portanti O. , Marcacci M. , Orsini M. , Barbria M.E. *et al* (2017) A novel Bluetongue virus serotype 3 strain in Tunisia, November 2016. Transboundary and Emerging Diseases 64, 709–715. 1‐7. 28299883 10.1111/tbed.12640

[vms3136-bib-0022] Tittarelli M . (2014) Standardized and validated for the detection of antibodies against the BTV in ruminants serum by Competitive Enzyme Linked Immunosorbent Assay. IZS, TE.

[vms3136-bib-0023] Vogtlin A. , Hofmann M.A. , Nenniger C. , Renzullo S. , Steinrigl A. , Loitsch A. *et al* (2013) Long‐term infection of goats with bluetongue virus serotype 25. Veterinary Microbiology 166, 165–173. 23834964 10.1016/j.vetmic.2013.06.001

[vms3136-bib-0024] Zientara S. , Viarouge C. , Höper D. , Beer M. , Jenckel M. , Hoffmann B. *et al* (2014) Novel bluetongue virus in goats, Corsica, France. Emerging Infectious Diseases 20, 2123–2125. www.cdc.gov/eid., Vol. 20, No. 12. 25418049 10.3201/eid2012.140924 PMC4257820

